# A spatial assessment of Nipah virus transmission in Thailand pig farms using multi-criteria decision analysis

**DOI:** 10.1186/s12917-019-1815-y

**Published:** 2019-03-04

**Authors:** Weerapong Thanapongtharm, Mathilde C. Paul, Anuwat Wiratsudakul, Vilaiporn Wongphruksasoong, Wantanee Kalpravidh, Kachen Wongsathapornchai, Sudarat Damrongwatanapokin, Daniel Schar, Marius Gilbert

**Affiliations:** 1 Department of Livestock Development (DLD), Bangkok, Thailand; 2 UMR 1225 IHAP, Université de Toulouse, INRA, ENVT, Toulouse, France; 3 Department of Clinical Sciences and Public Health, Faculty of Veterinary Science, Mahidol University, Nakhon Pathom, Thailand; 4 Food and Agriculture Organization of the United Nations, Regional Office for Asia and the Pacific, Bangkok, Thailand; 5 USAID Regional Development Mission Asia, Bangkok, Thailand; 6 Spatial epidemiology Lab. (SpELL), University of Brussels, Brussels, Belgium; 7 Fonds National de la Recherche Scientifique (FNRS), University of Brussels, Brussels, Belgium

**Keywords:** Nipah virus, MCDA, Thailand, Flying foxes, Risk-based surveillance

## Abstract

**Background:**

Thailand’s Central Plain is identified as a contact zone between pigs and flying foxes, representing a potential zoonotic risk. Nipah virus (NiV) has been reported in flying foxes in Thailand, but it has never been found in pigs or humans. An assessment of the suitability of NiV transmission at the spatial and farm level would be useful for disease surveillance and prevention. Multi-criteria decision analysis (MCDA), a knowledge-driven model, was used to map contact zones between local epizootic risk factors as well as to quantify the suitability of NiV transmission at the pixel and farm level.

**Results:**

Spatial risk factors of NiV transmission in pigs were identified by experts as being of three types, including i) natural host factors (bat preferred areas and distance to the nearest bat colony), ii) intermediate host factors (pig population density), and iii) environmental factors (distance to the nearest forest, distance to the nearest orchard, distance to the nearest water body, and human population density). The resulting high suitable areas were concentrated around the bat colonies in three provinces in the East of Thailand, including Chacheongsao, Chonburi, and Nakhonnayok. The suitability of NiV transmission in pig farms in the study area was quantified as ranging from very low to medium suitability.

**Conclusions:**

We believe that risk-based surveillance in the identified priority areas may increase the chances of finding out NiV and other bat-borne pathogens and thereby optimize the allocation of financial resources for disease surveillance. In the long run, improvements of biosecurity in those priority areas may also contribute to preventing the spread of potential emergence of NiV and other bat-borne pathogens.

## Background

Bat species have been identified as the reservoir of many pathogens infecting humans and animals. Pteropid bats (flying foxes) were found to be the natural host of the *Henipavirus* family that includes the Hendra virus (HeV) and the Nipah virus (NiV). HeV causes acute and highly fatal infection in humans and horses, was first described in Brisbane, Australia in 1994 [1]. Similar to HeV, NiV causes a range of clinical outcomes but primarily respiratory distress and encephalitis [2–4], and was first isolated from human patients and pigs in Malaysia in 1998 [5].

In tropical Asia, NiV is considered to be the main human and animal health concern among the viruses carried by bats. Even though flying foxes are found throughout tropical and sub-tropical Asia and Australia and on islands of the Indian Ocean and the western Pacific [6], NiV was mainly found in flying foxes with subclinical infections in tropical Asia (Malaysia, Cambodia, Thailand, Bangladesh, and India) [3, 7–10]. During the first NiV occurrence in Malaysia in 1998, pigs were found to be an amplifier. It is believed that NiV was passed from flying foxes to pigs and subsequently spilled over from pigs to other animals and humans [5, 11, 12]. Studies in Bangladesh also suggested that NiV may have passed directly from bats to humans without an amplification host [9, 13], and human-to-human transmission was observed in several outbreaks in Bangladesh and India [9–11].

In Thailand, NiV has been sampled from flying foxes since 2002 but no evidence of the virus in domestic animals has been found so far. Blood, saliva and urine samples of flying foxes (*P. hypomelanus, P. vampyrus*, and *P. lylei*) were collected during 2002–2004. All samples were tested in the Molecular Biology Laboratory for Neurological Diseases, Chulalongkorn University Hospital, with positive results to NiV presence and NiV antibody [8]. A longitudinal study was subsequently conducted on flying foxes (only *P. lylei*) between 2005 to 2007, which showed that two NiV strains previously identified circulating in Malaysia and Bangladesh were found in the bat’s urine [14]. The Department of Livestock Development (DLD) has been conducting surveillance of NiV infection in domestic pigs with serological and virological detection, and no NiV positive sample was ever found so far [15–17].

In the last few decades, Thailand’s pig production gradually increased with a continuing intensification of the sector [18]. The concentration was particularly marked in Thailand’s Central Plain because of its easy access to the major market of the Bangkok Metropolitan region [18]. Coincidentally, flying fox colonies are found mainly in the same areas [19–21], influenced by the availability of numerous water bodies (for drinking and releasing heat by dipping) and agricultural activities (for foraging) [21]. The region is also characterized by a fairly high human population density. Therefore, the regions surrounding the Bangkok metropolitan region appear as potential high-risk contact zones for NiV transmission among bats, pig and human population [18].

In a previous study, we used Potential surface analysis (PSA) as a first attempt to map suitable areas of NiV transmission to pigs in Thailand’s Central Plain [21]. The areas identified as higher suitability of NiV transmission were located around the Bangkok metropolitan area, covering 5417 km^2^ of 607 sub-districts, 125 districts, and 23 provinces [21]. However, the approach was limited by the somewhat arbitrary choices of weights of risk factors that were made along the process [21]. Furthermore, the approach did not allow incorporating risk factors at the farm level. The identification of relevant risk factors, their weights and the way they increase the risk spatially, or at the farm level, may be defined in a more explicit and thorough way in using a multi-criteria decision analysis approach [21, 22]. Therefore, the objectives of this study was to develop two complementary MCDA models, one aiming to map the suitability of NiV presence at the pixel level, and a second one aiming to quantify the suitability of NiV transmission at the farm level.

## Results

### Spatial model

The results of the decision making process are shown in Table 1. Seven spatial risk factors were identified by the experts: i) the bat preferred area, ii) the distance to the nearest bat colony, iii) the distance to the nearest forest, iv) the distance to the nearest orchard, v) the distance to the nearest water body, vi) the human population density, and vii) the pig population density. The results showed that, according to the experts, the distance to the nearest bat colony had the highest weight, followed by the pig population density, the bat preferred area, the distance to the nearest orchard, the distance to the nearest forest, and the distance to the nearest water body, respectively.

**Table 1:** Spatial risk factors, standardized methods, and relative importance of each factor

Factors	Fuzzy membership functions	Inflection points				Weights
		a	b	c	d	
Bat preferred area	Sigmoidal, monotonically increasing	0	0.5	0.5	0.5	0.18611
Distance to the nearest bat colony	Sigmoidal, monotonically decreasing	5 km.	5 km.	5 km.	30 km.	0.28016
Distance to the nearest forest	Linear, monotonically decreasing	100 m.	100 m.	100 m.	23 km.	0.06887
Distance to the nearest orchard	Linear, monotonically decreasing	0	0	0	500 m.	0.11719
Distance to the nearest water body	Linear, monotonically decreasing	0	0	0	1 km.	0.03663
Human population density	Sigmoidal, monotonically decreasing	100 per km^2^	800 per km^2^	800 per km^2^	800 per km^2^	0.06979
Pig population density	Linear, monotonically increasing	0 per km2	1,000 per km2	1,000 per km2	1,000 per km2	0.24126

**Fig. 1:**
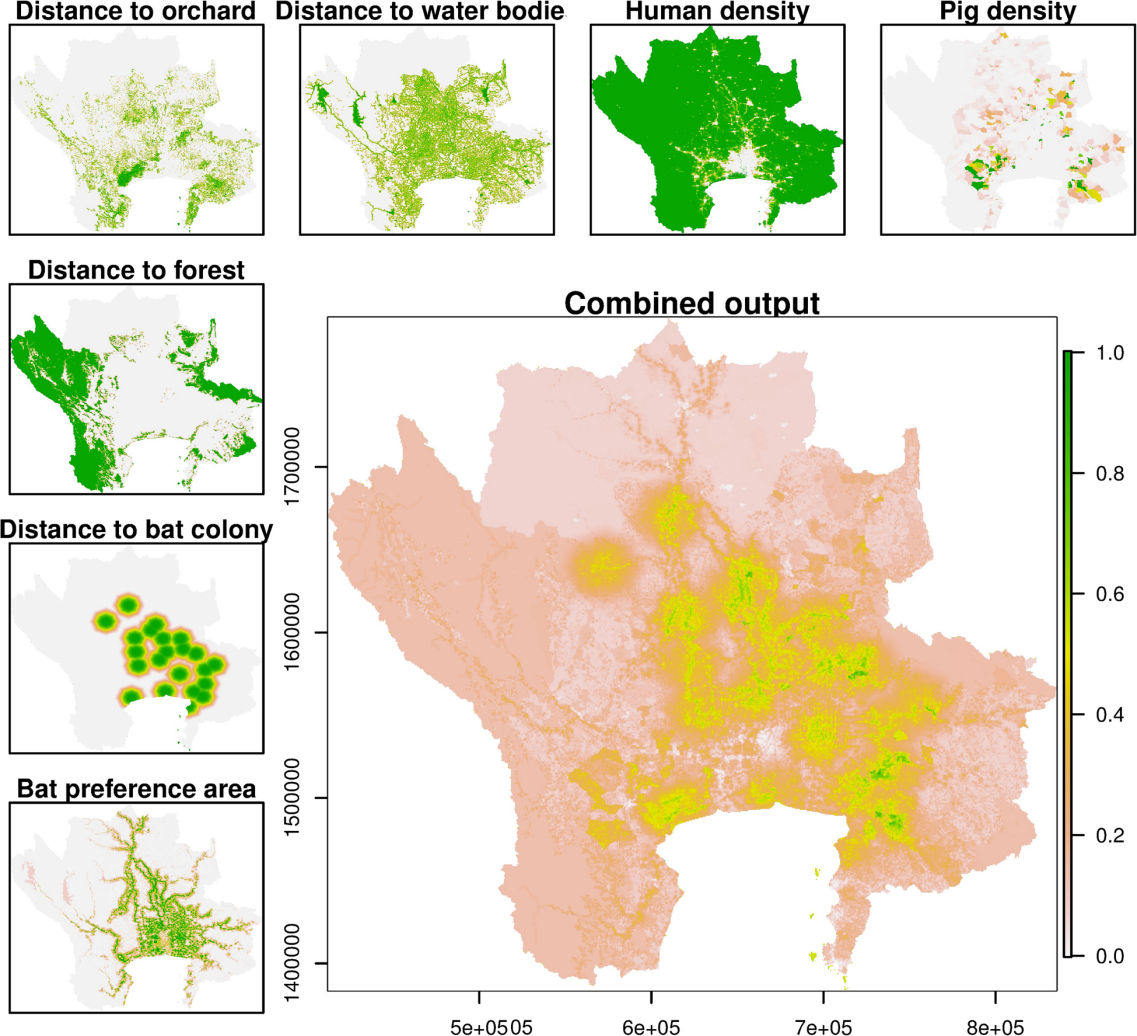
Standardized factors and suitability map of NiV transmission in pigs in the Central Plain of Thailand. The standardized factors (from the left bottom) including bat preferred area, distance to the nearest bat colony, distance to the nearest forest, distance to the nearest orchard, distance to the nearest water body, human population density, and pig population density. The large map shows the final combined suitability map

The resulting high-suitability areas were clustered nearby the bat colonies, but varying levels of risk could be observed depending on their surrounding (Fig. 1). The high-suitable areas, defined as those with a scale > 0.6 were extracted and aggregated the district level that would make them more conveniently usable by local veterinary officers. Twenty-seven provinces contained at least one pixel ranked as high-suitability, but this number was reduced to 18 provinces (101 districts and 496 sub-districts) by removing high-suitability areas covering less than 1 km^2^. The three provinces in the East of Thailand, including Chacheongsao, Chonburi, and Nakhonnayok, were found to be the one with the largest areas of high suitability of NiV transmission.

**Fig. 2:**
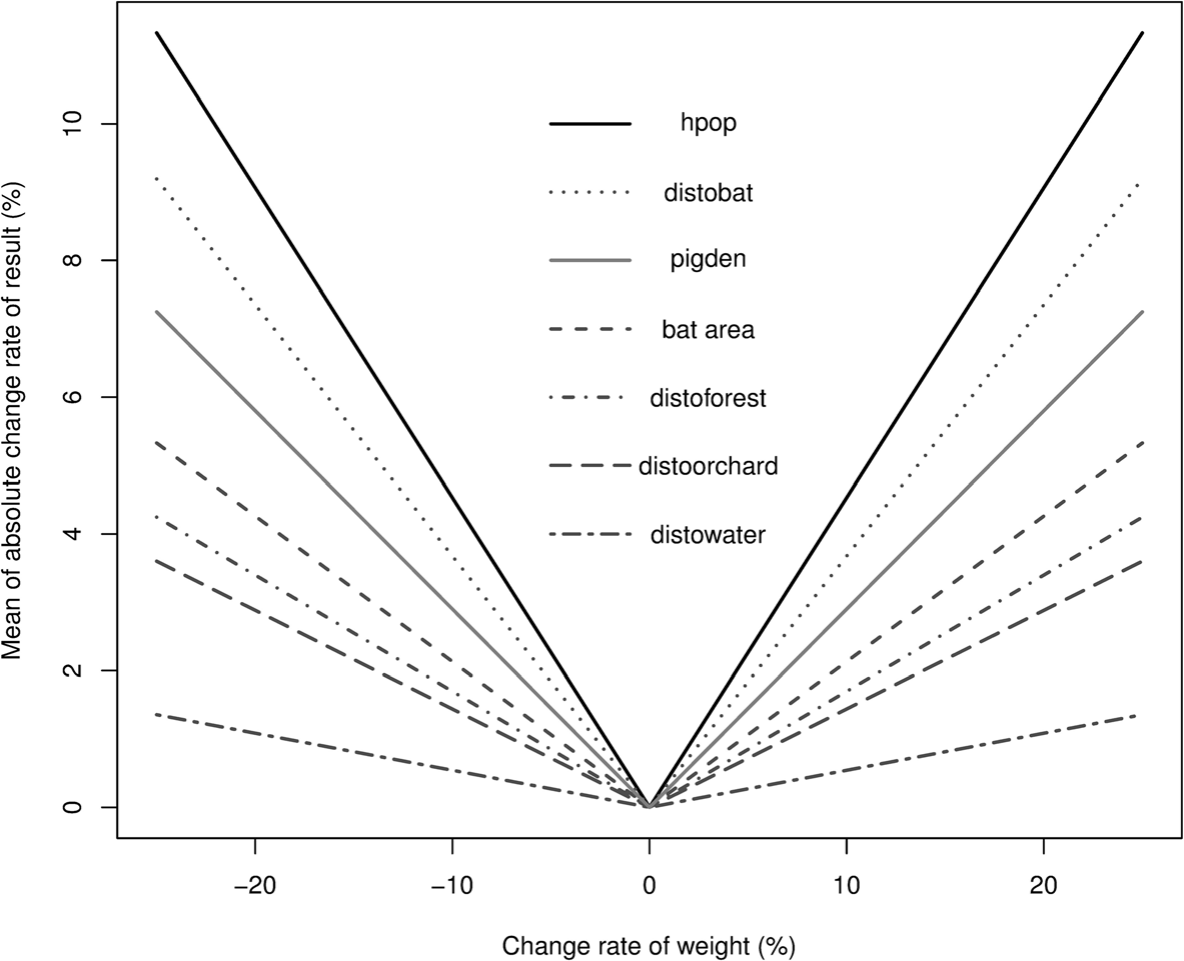
Mean absolute values of the change rate (MACRs) for the suitability maps under simulations. (hpop: human population density, distobat: distance to the nearest bat colony, pigden: pig population density, batarea: bat preferred area, distoforest: distance to the nearest forest, distoorchard: distance to the nearest orchard, and distowater: distance to the nearest water body)

For the OAT sensitivity analysis, the simulated suitability maps for NiV transmission in pigs in the Central Plain of Thailand were generated with the weight of each factor changed from − 25 to 25% with a step size of 1%. The MACRs were used to display the sensitivity of each factor, which a high gradient indicates a greater change in values of the output maps (high sensitivity). As a result (Fig. 2), the outputs were most sensitive to human population density, followed by the distance to the nearest bat colony, the pig population density, the bat preferred area, the distance to the nearest forest, the distance to the nearest orchard, and the distance to the nearest water body.

**Fig. 3:**
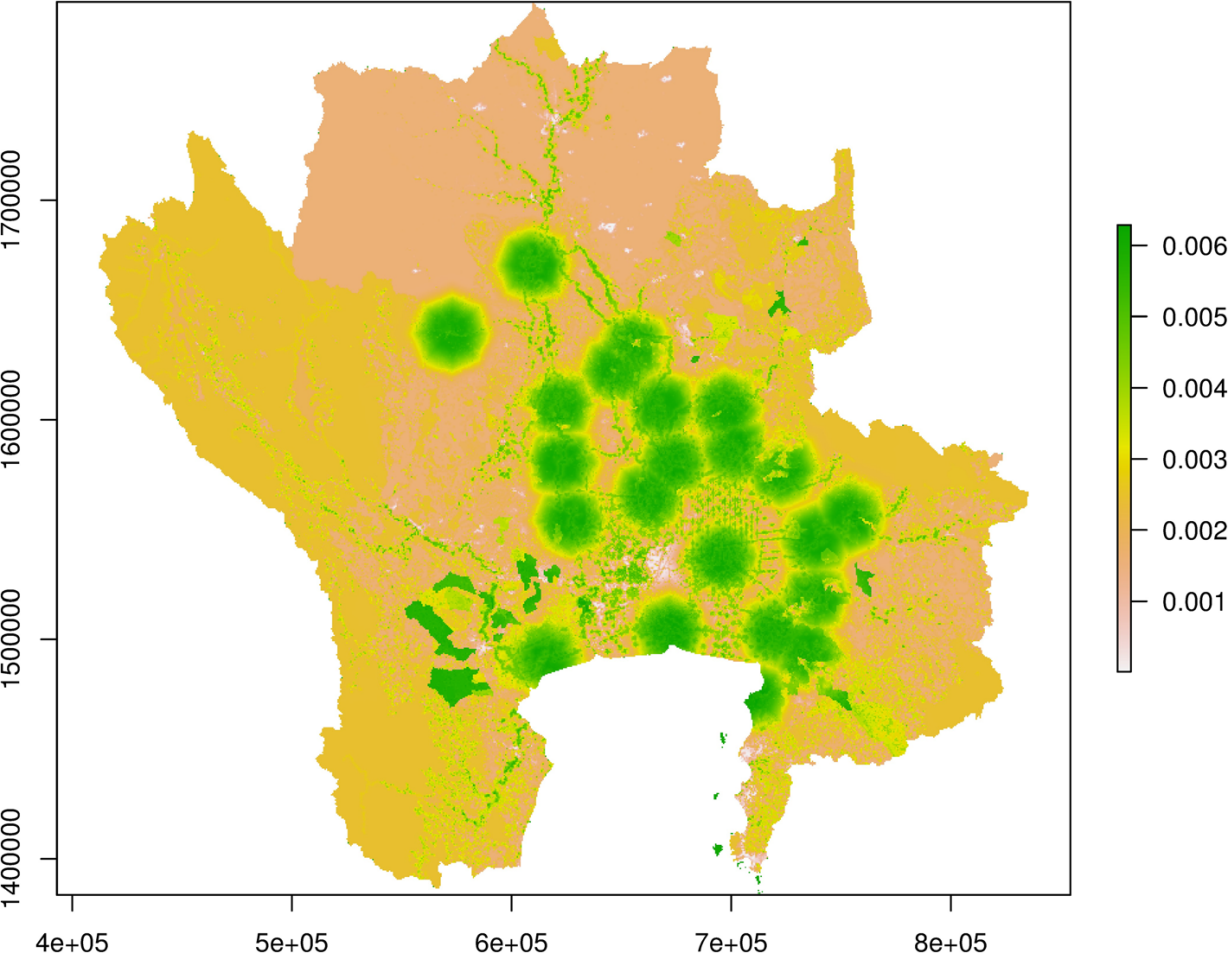
Uncertainty map. The map shows standard deviation of the suitability maps for NiV transmission in pigs in the Central Plain of Thailand

The uncertainty analysis showed a fairly robust result and a spatial heterogeneity. The uncertainty surface remained stable with the maximum standard-deviation value (STD) being less than 0.1 (Fig. 3) even through risk factors were varied. This implies that the predicted suitability areas for NiV transmission in pigs in the Central Plain of Thailand according to the suitability index are fairly robust. The result also showed a spatial heterogeneity in uncertainty, with higher uncertainty in high suitability areas of NiV transmission in pigs.

### Farm model

**Fig. 4:**
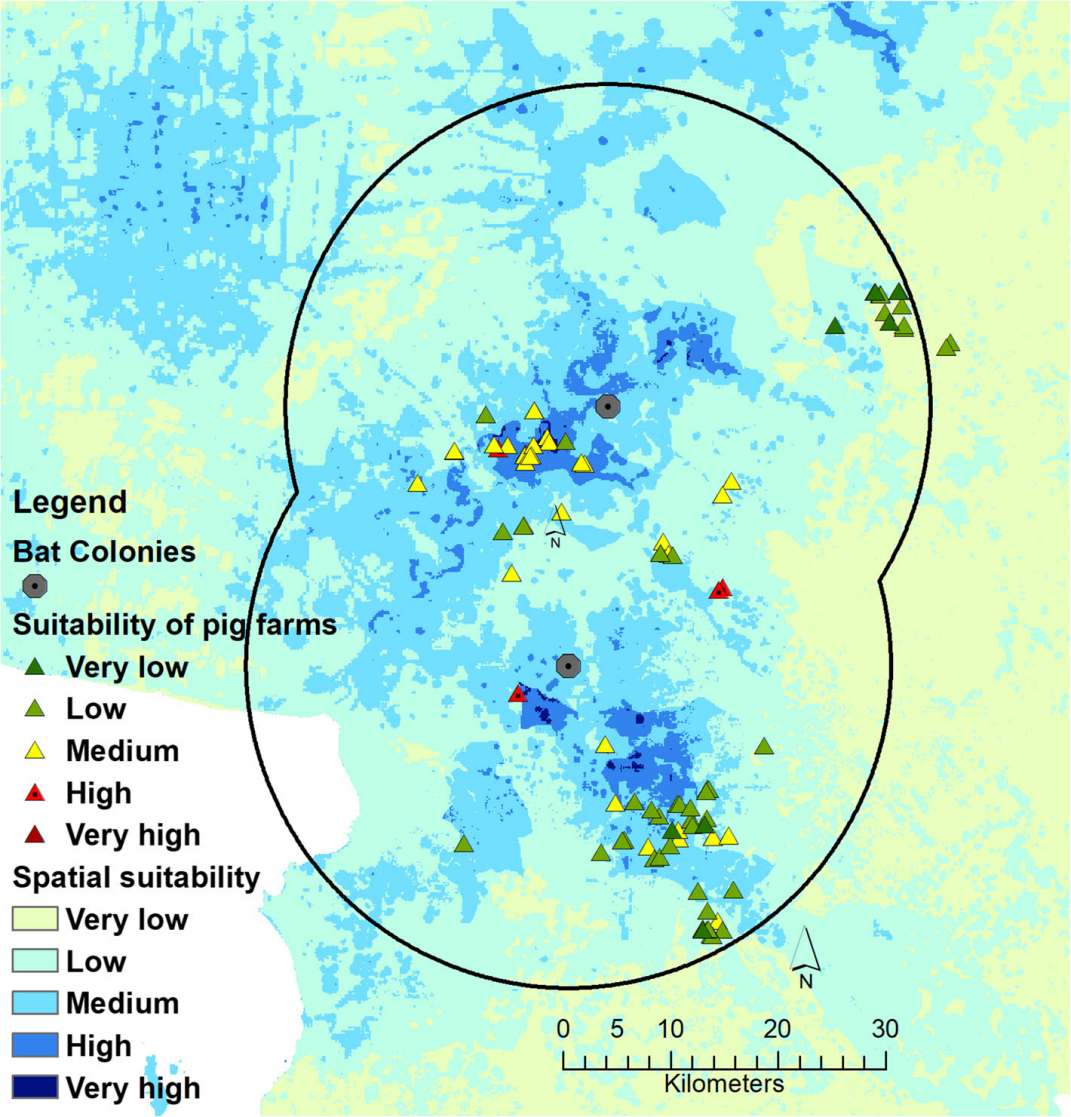
The suitability of NiV transmission in spatial and farm level in study area. Map shows the suitability levels of 89 pig farms (from farm model) within a 30-km radius surrounding two bat colonies in the East of Thailand overlayed on the spatial suitability of NiV transmission (from spatial model)

The results of the decision making process are shown in Table 2, which details the risk factors, their scale and weight. The experts defined twelve risk factors that would be important at the farm level. The three most important identified factors were the type of pig house, and the presence and frequency of flying foxes found in the farm area. The final score of each farm was from the WLC method, categorized in 5 suitability levels (< 1.5 = very low, > 1.5–2.5 = low, > 2.5–3.5 = medium, > 3.5–4.5 = high, and > 4.5 = very high). As shown in Fig. 4, there were 7 farms with very low suitability, 46 farms with low suitability, 30 farms with medium suitability, and 6 farms with high suitability. The mean score of the 89 farms was 2.374 with a standard deviation of 0.721. The breakdown of suitability at the farm level and by farm size is presented in Table 3. One can note that the high suitable farms were small-scale (3 farms, < 500 pigs/farm) and medium-scale farms (3 farms, 1000–5000 pigs/farm), and that the majority of farms were ranked in the low (46) and medium (31) suitability.

**Table 2:** Risk factors in farm level, standardized methods, and relative importance of each factor

Factors	Very low (1)	Low (2)	Medium (3)	High (4)	Very high (5)	Weight
Certified standard farm	Yes		Submitted but not certified		Not certified	0.05652
Type of pig house	Close (evaporation)	Close (wind tunnel)	Open (net)		Open (no net)	0.22995
Flying fox bat (s) found in farm area	Never		A bat flying through	A bat eating in farm	A bat roosting in farm	0.13653
Frequency of flying fox bat (s) found in farm area	Never	Seldom (<12 times per year)	Occasionally (at least a time per month)	Often (> 2 times per week)	Always (almost every day)	0.13653
Presence of fruit trees in farm	No		< 20 trees		>20 trees	0.06654
Number of fruit trees less than 15 m. away from the pig house	No			<5 trees	>5 trees	0.10251
Presence of high trees in farm	No	<20 trees		>20 trees		0.03272
Number of high trees being less than 15 m. away from pig house	No		<5 trees	>5 trees		0.06367
Type of other animals in farm	No	Others	Cow/horse/goat	Dog/cat	Rodent	0.02538
Presence of fence surrounding farm	Yes			No		0.02643
Quarantine of at least 14 days before introduction of new pig	Yes			No		0.02643
Spatial suitability of NiV transmission where this pig farm is located	Very low	Low	Medium	High	Very high	0.09678

**Table 3:** Suitability levels of pig farms classified by farm size

Farm size (Pig/farm)	Suitability levels					
	Very low	Low	Medium	High	Very high	Total
	(<1.5)	(1.5–2.5)	(2.5–3.5)	(3.5–4.5)	(>4.5)	
<100	0	1	1	2	0	4
100–500	3	8	6	1	0	18
500–1000	1	21	10	0	0	32
1000–5000	3	15	11	3	0	32
>5000	0	1	2	0	0	3
Total	7	46	30	6	0	89

## Discussion

GIS-based MCDA was used in this study to evaluate the suitability for NiV transmission in pigs in Thailand in the absence of actual data in NiV occurrence in pigs. Data-driven models are far more frequently used to infer risk and to quantify the association between an event of interest (such as disease occurrences) and explanatory variables by using statistics [23]. For example, autoregressive logistic regression models and boosted regreesion tree models have been used to model the distribution of Highly Pathogenic Avian Influenza (HPAI) H5N1 [24–27] and Porcine Reproductive and Respiratory Syndrome (PRRS) in Thailand [28]. However, as NiV has never been reported in pigs or domestic animals in Thailand, data-driven models could not be applied. With the present situation, NiV exists in the country [8] and the neighboring countries [3, 7, 9, 10] carried by the flying foxes; therefore, the prevention of NiV transmitted to pigs should be immediately implemented by targeting the specific areas and farms with high chances of NiV occurrence. Knowledge-driven models such as Potential surface analysis (PSA) or MCDA provide an interesting alternative to model the suitability of NiV distribution in space, or at the farm level as a way to prioritize surveillance and improve prevention [8]. Knowledge-based models have been found to provide fairly good accuracy metrics in previous studies [29], but these somewhat benefited from several years of experience and published papers where data-driven approaches had been used to identify important risk factors. So, in situations where very little is known about a disease and its main risk factors, their outputs help converting the current state of knowledge into a visualisation where all factors are combined together, but the quality of the predictions may be compromised by the misidentification of some unknowns factors.

In this regard, the current lack of epidemiological knowledge on NiV in pigs is a limitation of this study. The two possible sources of a priori *knowledge* include literature review and experts’ opinion [30, 31] to identify spatial risk factors. One should note that those two sources are rarely independent, because the expert opinion can be influenced by their knowledge of the published litterature, and the litterature is itself produced by experts. The only litterature on NiV in Thailand concerned bats [8, 32, 33], so there was little experience of the experts with what may be applicable to the country, and the national expert views would have been mostly informed by knowledge gained from epidemiological reports from other countries where NiV was found. The subjectivity associated with experts’ choices in the consideration of risk factors, membership functions and weights also has indeed been often identified as an important limitation of the MCDA approaches [30, 31]. Furthermore, map validation could not be applied in this study in the absence of presence data [30].

The geographical conditions found in the Central Plain of Thailand are favourable not only to livestock development but also provide good habitats for flying foxes’ colonies. The Central Plain is recognized as the major feed production area in Thailand, with an abundant supply of rice bran and broken rice, low transport cost and short shipment time to the major consuming centre (Bangkok) as well as ports for export [18]. It is a large and fertile area able to supply a dense human population [34], with flat landscape and a well-developed irrigation system for wet-rice agriculture. Metropolitan Bangkok, the focal point of trade, transport, and industrial activity, is situated on the southern edge of the region at the head of the Gulf of Thailand [34]. The livestock industry has therefore developed particularly in these areas, supplying both national and international markets. Large-scale pig farms found in the suburban areas surrounding the main cities, particularly around the Bangkok Metropolitan region [18] are mainly commercial (breeding and fattening) pig farms. A similar spatial pattern was previously reported for poultry [35], where most of the intensively production units are concentrated around Bangkok. Unfortunately, flying fox colonies are also found mainly on Thailand’s Central Plain, particularly in locations surrounded by bodies of water (for drinking and cooling down), vegetation (for foraging), and safe havens such as Buddhist temples (for survival from human hunting) [21]. So, intensification of pig production in this particular region over time may have created favorable conditions for transmission of NiV to pigs.

Spatial risk factors of NiV transmission in pigs identified by experts in this study were associated with three components of the landscapes including i) natural host (bat preferred area and distance to the nearest bat colony), ii) intermediate host (pig population density), and iii) environment (distance to the nearest forest, distance to the nearest orchard, distance to the nearest water body, and human population density). The bat preferred area (potentially suitable sites) was mapped according to the assumption that bat colonies may move from one site to another [21]. The distribution of flying fox colonies is dynamic and changes have been observed over time [19–21], caused by different factors such as hunting, damage of roosting trees, invasion by other species, or simply to expand the sizes of the colony [21]. A short distance to bat colonies was identified as a particularly important risk factor in this study. Foraging individuals commuting between day roosts and foraging areas each night were studied using high-resolution global positioning system (GPS) loggers, and these studies showed that the maximum linear distances covered during a night varied greatly between individuals, ranging between 2.2 and 23.6 km [36]. The study also reported that tracked bats mostly foraged in farmland, plantations, small mangrove remnants, and gardens [36]. This justified the distance to the nearest forest and the distance to the nearest orchard as factors to be considered in this analysis. The distance to the nearest water body was considered based on the behavior of bats that need water for drinking [37, 38] and that have been observed cooling down in large water bodies on hot days [39, 40]. The human population density was considered as a spatial factor because the flying foxes are likely to forage away from high human density area caused by hunting [21]. A pig population density was also included in the study. A detailed investigation carried out in Malaysia indicated that NiV epidemics could have been caused by intensifying agriculture (pig and mango production) in the country [5, 12, 41].

Improvement of biosecurity at the farm level can strongly reduce the risk of animal-to-pig transmission. The risk factors at the farm level identified by experts were related to three possible sources of NiV including flying foxes, infected pigs, and infected other animals such as dogs, cats, goats, sheep and horses. This was based on investigation of the index case (first occurrence case) in Malaysia reporting that pigs were first infected by NiV shed by flying foxes through their urine or partial-eaten fruits [12]. Further investigation showed the virus to subsequently spill over from pigs to other animals and humans via respiratory droplets or close contact [5, 11]. Therefore, such infected pigs (from other farms) and infected other animals (both inside and outside farm) may possibly transmit NiV to pigs as well. So, avoiding interface between pigs and the possible sources by improving biosecurity at the farm level could prevent bat-to-pig, pig-to-pig, and animal-to-pig transmission. Several example of measures could be considered such as i) the use of nets to cover pig houses to prevent the contact between pigs and flying foxes, ii) the cutting down of fruit trees that are nearby the pig houses to reduce farm attractivity, iii) the quarantine and observation of new pigs before they enter the pig houses, or iv) the use of fence to prevent other animals from entering the pig farm.

Although suitability evaluation of pig farms in the study area mostly showed farms classified as big at very low to medium suitability, some pig farms had higher suitability scores. The fact that the majority of pig farms in the study area (93.3%) had very low to medium suitability of NiV transmission is in fact not so surprising. The area corresponds to the foot and mouth disease (FMD) free-zone of Thailand [16], where strong surveillance of FMD gives access to international markets. FMD is indeed an important trade barrier due to its high capacity to be transmitted to a wide range of hosts including cattle, pigs, goat, sheep, and other ruminants with a high economic impact [42]. Pig production intensified in Thailand, with a shift from extensive production systems (raise small amount of pigs for household consumption and additional income) towards more intensive production systems (commercial purpose) [18]. These changes were particularly marked in Eastern Thailand where the predominance of intensive pig production systems promoted the establishment of the FMD-free zone to gain access to international markets. However, in order to gain the FMD-free zone status, most pig farms in this part of Thailand have been improving their biosecurity and these provinces now contain a lower proportion of small intensive farms than many other Thailand provinces. Our results showed 6 pig farms (6.7%) presenting a high suitability, with 3 small-scale farms (< 500 pigs per farm) and 3 medium-scale farms (between 1000 and 5000 pigs per farm). These may need further investigation and possible increased surveillance and biosecurity measures.

The farm-level evaluation of NiV suitability was only performed in limited areas, and as our results demonstrate, these could be expanded to other parts of Thailand’s Central Plain in order to have a more comprehensive overview of the farms at risk. In this regard, the results of the spatial model may be particularly useful, as they may guide the deployment of farm-level surveys in other areas. Eighteen provinces with 101 districts and 496 sub-districts were identified as high-suitability areas of NiV infection by the spatial model. Farm-level suitable quantification using questionnaire developed on mobile (phone or tablet) applications could be implemented by firstly focusing on all pig farms located in the high suitable districts and then expanding to medium and low suitable districts. In addition, since several of the risk factors are somewhat generic (e.g. presence of fence, quarantine of entering animals), this may allow having a broader overview of risk factors at the farm level that could be relevant to other diseases. In this regard, the addition of other risk factors relevant to other diseases may be added to the questionnaire. This would also present several opportunities to inform the farmers on the way to improve biosecurity and reduce the risk of NiV infection, but also of other diseases such as FMD or PRRS. Biosecurity is the implementation of measures that reduces the risk of the introduction and spread of disease agents [43], or in other words, the “keep microbes away from pigs” and/or “keep pigs away from microbes” [43]. One should note that biosecurity does not necessarily reflect the same practical measures in high-income countries, where it mostly correspond to large investments in infrastructures and equipment, than in low or middle income countries, where biosecurity improvements in the smallholder sector can already be achieved through very simple and low-cost precautionary measures [44].

## Conclusions

The spatial and farm suitability models developed here could be immediately applied to implement risk-based surveillance and to improve biosecurity in the long run. The implementation of risk-based surveillance with prioritized areas and farms may increase the chances of detecting NiV and other bat-borne pathogens in pig farms, and also reduce the financial burden of animal disease surveillance [45]. So far, the targeted surveillance implemented by the Department of Livestock Development (DLD) has been focusing on areas according to their pig density [16], but this may have missed the critical influence of other factors. The MCDA approach allows to integrate these different factors in a transparent and explicit way, allowing to account for components associated with the natural host, the intermediate host, and the environment. So, for a constant amount of resource in surveillance, this may increase the chance of NiV detection in comparison to the current scheme.

## Methods

### Data collection

**Fig. 5:**
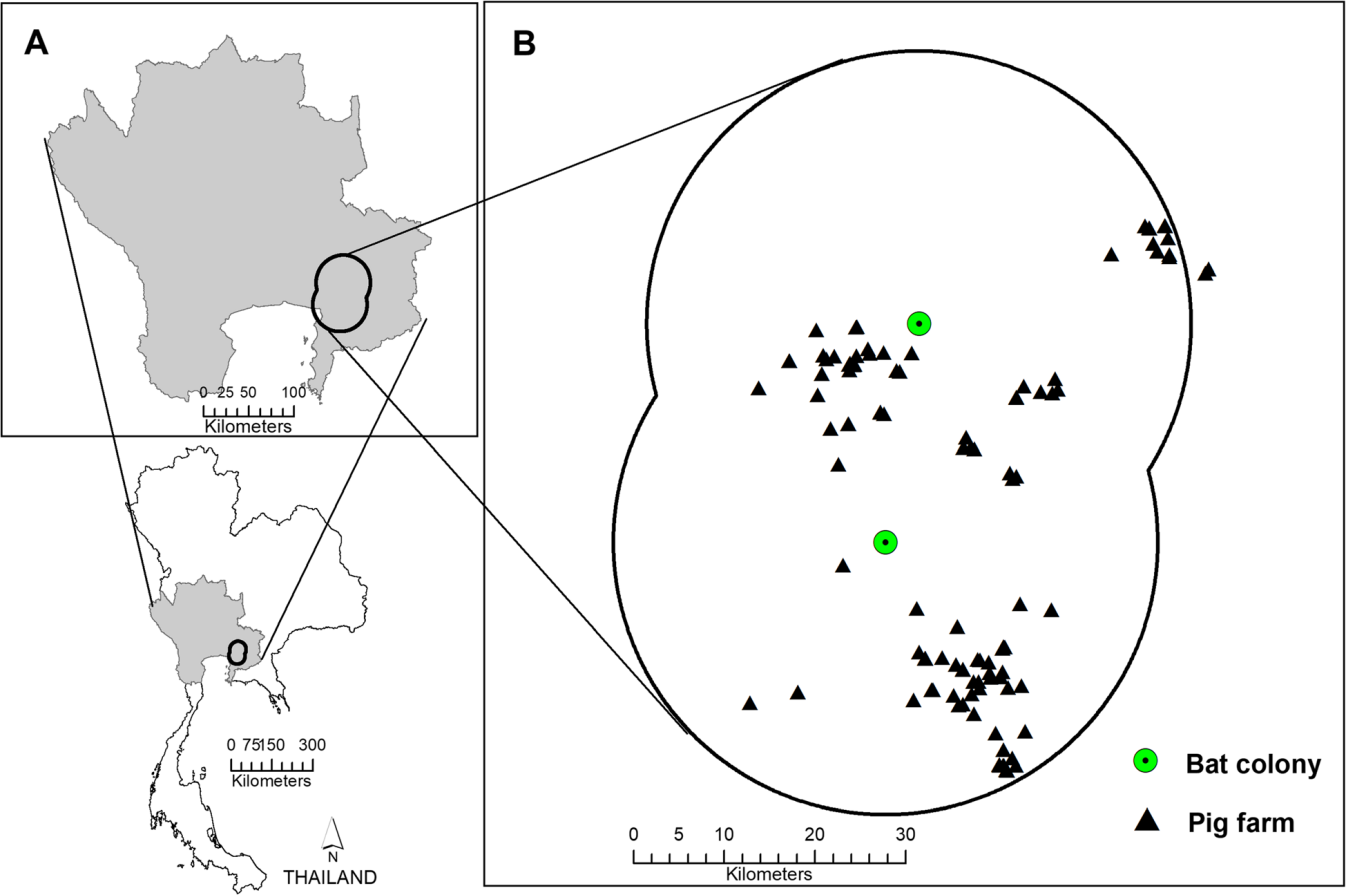
Study area for suitability evaluation of NiV transmission in pigs. **a** Study areas of the spatial model covering 93,826.2 km^2^ of 27 provinces across western, central, and eastern Thailand and (**b**) of the farm model, including a 30-km radius surrounding two bat colonies: Wat Luangprommawat (lower gray dot) and Wat Phobangkla (upper gray dot)

The analysis of suitability of NiV transmission in pigs in Thailand was analyzed at the level of 100- m pixel on one hand (spatial model), and of the farm on the other hand (farm model). The spatial model was based on spatial risk factors that could influence the distribution of NiV and therefore determine the suitability of NiV transmission in pigs. The study area for the spatial model included 27 provinces of western, central and eastern Thailand, covering a total area of 93,826 km^2^ (Fig. 5a). The area was selected following previous studies [19–21] reporting colonies of flying foxes belonging to two species: the Lyle’s flying fox (*P. lylei)* living in central Thailand, and the Large flying fox or Greater flying fox (*P. vampyrus)* living along the coast of eastern Thailand. Recently, there were 22 colonies reported in these areas [21].

The primary data used in this study are listed in Table 3. The bat preferred areas were modeled in a previous study using Ensemble modeling (EM) by Thanapongtharm et al (2015) [21]. Distance risk factors were processed in ArcGIS 10.2 with the cost distance tool, and the objects from which the nearest distances were estimated included the bat colonies, forests, orchards, and water bodies. The bat colonies’ coordinates were provided by Department of National Park, Wildlife and Conservation [46] whereas the vector data of forests, orchards, and water bodies distribution were provided by Land Development Department (LDD) [47]. The human population density raster data set at 100-m resolution was obtained from the Worldpop project [48]. For pigs, we used the pig population density modeled using a Random Forest (RF) by Thanapongtharm et al (2016) [18]. All geographical data were converted in raster data sets with a 100-m resolution to match the resolution of the finest data set. In this step, ArcGIS 10.2 software was used to manage all geoprocessing.

For the farm model, risk factors at the farm level such as biosecurity measures and characteristics of farms’ environments were used to evaluate the suitability of NiV infection. We selected 89 pig farms (25%) out of a total of 359 pig farms located in a 30-km radius surrounding two bat colonies in the East of Thailand: Wat Luangprommawat in Chonburi province and Wat Phobangkla in Chacheongsao province (Fig. 5b). This radius was determined based on previous knowledge of bats foraging behaviors. Using high–resolution GPS loggers, Weber et al (2015) showed that maximum linear distances between day roosts and foraging areas for flying foxes at these two temples ranged between 2.2 and 23.6 km [36]. Pig farms within 30 km were selected with criteria as follows: i) farms with low biosecurity, ii) open house system, and iii) there were orchards close to the pig house or farm.

The defined factors used to carry out this study are listed in Table 2. We designed questionnaire on mobile application and used it to evaluate the suitability of pig farms to NiV transmission which were located in the study area. Farmers were provided all of the information required regarding their participation in the study to obtain their informed consent.

### Data analysis

In the absence of NiV positive found in pigs through surveillance that would allow data-driven approach, an alternative to map the suitability of infection through the integration of several risk factors is to use knowledge-driven models such as Multi-criteria Decision Analysis (MCDA). MCDA is a static knowledge-driven model which aims to rank best choices by defining a set of weighted rules based on existing published and/or expert knowledge [23]. Many MCDA methods have been developed since 1960s [49] including the Multi-Attribute Utility Theory (MAUT), outranking (PROMETHEE and ELECTRE), and the Analytical Hierarchy Process (AHP), the later having significantly expanded in uses over the last decade [22].

**Fig. 6:**
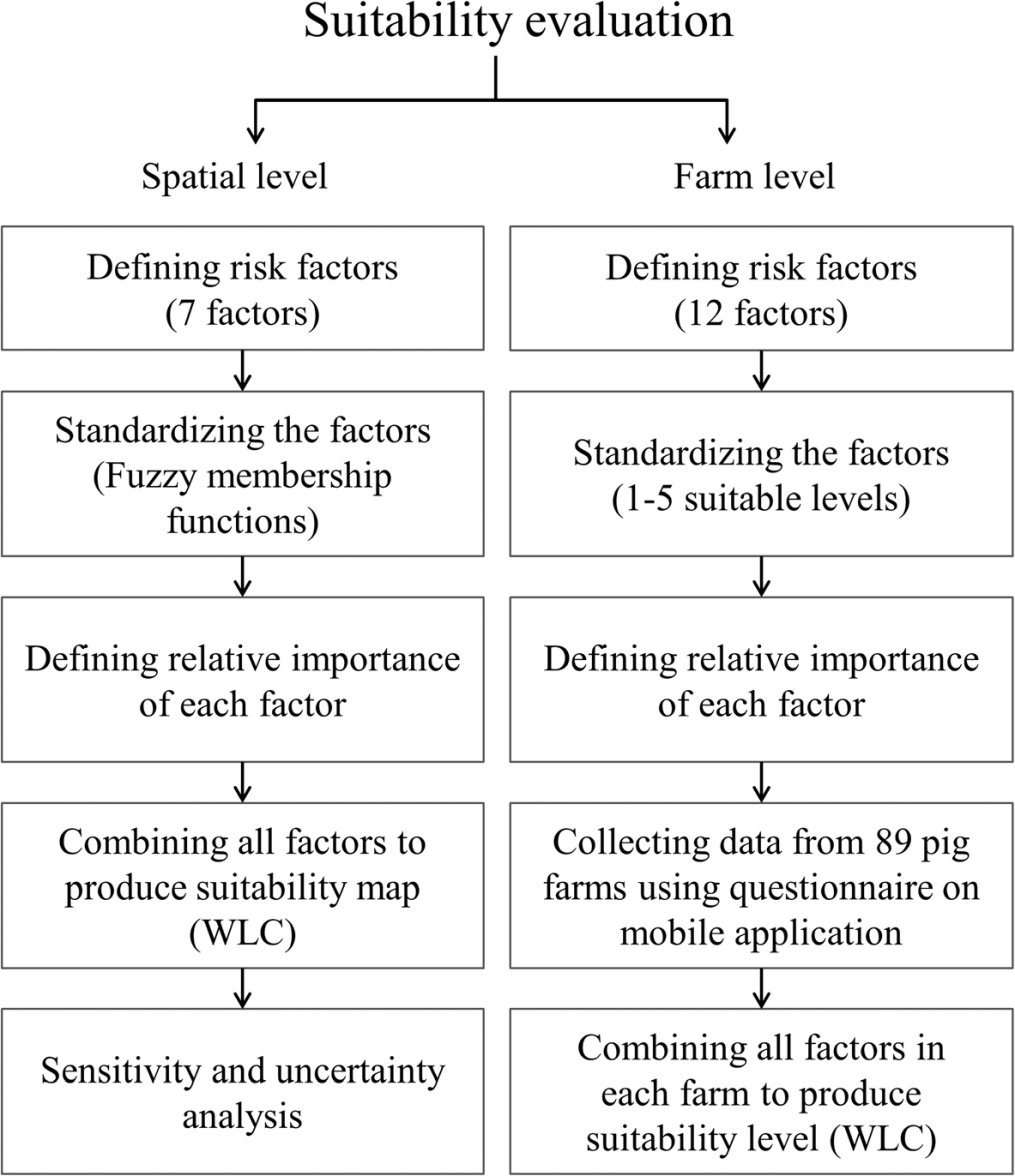
Diagram of the suitability evaluation process followed for the spatial and the farm models

AHP was used in the present study, for its power and simplicity [22, 50], and involved the implementation of the following sequence of analytical steps [23]: 1) the definition of risk factors; 2) the standardization of factors; 3) the definition of the relative importance of each factor; 4) the combination of all factors and constraints to produce a final weighted estimate of suitability; and 5) a sensitivity and uncertainty analysis (Fig. 6). The decision making part of the analytical procedure (step 1–3) was implemented in Microsoft excel, while their implementation in term of spatial distribution was performed using R (Raster package [51]), IDRISI [52] and ArcGIS 10.2 [53].

### Definition and standardization of the risk factors

A workshop was organized to go through the decision-making process, which was attended by 20 experts in epidemiology, virology, pig farming systems, and bats’ ecology. The experts were divided into two groups, for the spatial and farm models, respectively.

Factors were standardized so that they could be compared. For the spatial model, the experts defined the relationship between the values of each factor and the suitability of NiV distribution as ranging from 0 (unsuitable) to 1 (highly suitable) by using fuzzy membership functions. Four types of relationships were proposed to the experts: linear, sigmoidal (s-shaped), j-shaped, and user-defined, with increasing, decreasing, or symmetric functions [30]. The FUZZY tool from the IDRISI software was used to implement this standardization step. The FUZZY tool requires the position along the X axis of each risk factor of 4 parameters (a, b, c, and d) governing the shape of the fuzzy membership function [54]. For the risk factors of the farm model, the experts defined the relationship between each of the factors and suitability using a 6 points scale: 0: constraint (i.e. not suitable at all), 1: very low, 2: low, 3: medium, 4: high, and 5: very high).

### Definition the relative importance of each factor

To define the relative importance of each factor, a pairwise comparison technique was used. The procedure consisted in comparing each pairs of factors by a nine-point continuous comparison scale (Table 4). Experts first assigned their scores for all pairwise comparisons individually, and then assigned the final scores altogether. The weight value for each factor (Wi) was calculated by taking the eigenvector corresponding to the largest eigenvalue of the pairwise score matrix, and then normalizing the sum of the components to a unity [55–57]. The consistency of the matrix was verified using consistency ratio (CR), which was calculated as the consistency index (CI) divided by random index (RI). CI is calculated as:

**Table 4:** Nine-point scale values used in the pairwise comparison of factors

Intensity of importance	Description
1	Equal importance
3	Moderate importance
5	Strong or essential importance
7	Very strong or demonstrated importance
9	Extreme importance
2,4,6,8	Intermediate values
Reciprocals	Values for inverse comparison

1 $$ CI=\frac{\lambda_{max}-n}{n-1} $$

where λ_max_ is the maximum eigenvalue of the judgement matrix and n is the number of factors. RI is derived from Saaty (1980) [58] and is entirely dependent on the number of factors in the analysis (Table 5). If CR is higher than 0.10, then some pairwise values need to be reconsidered and the process is repeated until the desired value of CR less than 0.10 is reached [59].

**Table 5:** Saaty’s random index (RI)

No of factors	1	2	3	4	5	6	7	8	9	10	11	12	13	14	15
RI	0.00	0.00	0.58	0.90	1.12	1.24	1.32	1.41	1.46	1.49	1.51	1.54	1.56	1.57	1.58

### Combination of all factors to produce a final weighted estimate of suitability

Weighted Linear Combination (WLC) [60] method was used to combine all factors and constraints to generate the suitability maps (spatial model) and risk level of each pig farm (farm model). This method produces a final weighted estimate of suitability for each location in the study area. In the WLC, each standardized factor is multiplied by its corresponding weight, these are summed, and then the sum is divided by the number of factors. Its equation is as:

2 $$ S=\sum \limits_{i=1}^n{w}_i{x}_i{c}_j $$

where *w*_*i*_ is the weight of criteria i, *x*_*i*_ is the criterion score of criteria i (value of corresponding the raster cell in the criterion raster map), *n* is the number of criteria, *c*_*j*_ is the criterion score (1 or 0) of constraint j.

For the spatial model, WLC was implemented using IDRISI software (Multi-criteria evaluation tool) to produce a final weighted estimate of suitability for each pixel in the study area. For the farm model, we combined all factors and constraints using WLC to produce a final weighted estimate of suitability of each pig farm.

### Sensitivity and uncertainty analyses

Sensitivity Analysis (SA) was implemented by applying One-At-a-Time (OAT) method, which works by changing one input factors at a time and evaluating the effect of the change on the output [61]. Even though there are three most commonly used ways to measure the sensitivity, by changing the values of the criteria, by changing the relative importance of criteria, and by changing the weights of the criteria, we only investigated the later. We choose the OAT method for its simplicity and good comparability results.

The SA was performed for each objective using a previously proposed framework [56], whereby two parameters are set: a step size of 1% and a range of 50% (± 25%). By changing one factor at a time, all other factor can be fixed, at least to a great extent, to their central or baseline value. The sum of all criteria weights at any percent change (PC) level should always be equal to 1. The weight of the main changing criterion (*W*(*c*_*m*_, *pc*)) at a certain PC level can be calculated as

3 $$ W\left({c}_m, pc\right)=W\left({c}_m,0\right)+\left(W\left({c}_m,0\right)\ast pc\right),\kern1em 1\le m\le n $$

where *W*(*c*_*m*_, 0) is the weight of the main changing criterion *c*_*m*_ at the base run (the original weights). The weights of the other criteria *W*(*c*_*i*_, *pc*) are adjusted proportionally in accordance with *W*(*c*_*m*_, *pc*) in order to maintain the sum of all criteria weights at any PC of 1, with the equation is

4 $$ W\left({c}_i, pc\right)=\left(1-W\left({c}_m, pc\right)\right)\ast \left(\frac{W\left({c}_i,0\right)}{1-W\left({c}_m,0\right)}\right),\kern0.75em i\ne m,1\le i\le n $$

where *W*(*c*_*i*_, 0) is weight of the *i*-th criterion *c*_*i*_ at the base run.

We evaluated this step using the mean of the absolute change rate (MACR) [62]. In each simulation, the original suitability map (the original weights) and the output map of the alternative model (changing criterion weights) were quantitatively matched through a pixel-by-pixel comparison. The MACR was calculated by the following equation:

5 $$ MARC\left({w}_J, cr\right)=\sum \limits_{k=1}^N\frac{1}{N}\times \left|\frac{R_k\left({w}_j, cr\right)-{R}_0}{R_0}\right|\times 100\% $$

where *MARC*(*w*_*J*_, *cr*) is the mean absolute value of the change rate with ${\overline{w}}_J$ as a change rate, and N is the number of pixels. In addition, an uncertainty surface resulting from the changes in weights was produced for the study area, representing the standard deviation of the different suitability maps [30, 63].

## Abbreviations

AHP: Analytical Hierarchy Process
DLD: Department of Livestock Development
EM: Ensemble modeling
FMD: Foot and mouth disease
HeV: Hendra virus
HPAI: Highly pathogenic avian influenza
LDD: Land Development Department
MACR: Mean of the absolute change rate
MAUT: Multi-Attribute Utility Theory
MCDA: Multi-criteria decision analysis
NiV: Nipah virus
OAT: One-At-a-Time
PC: Percent change
PRRS: Porcine Reproductive and Respiratory Syndrome
PSA: Potential surface analysis
RF: Random forest
SA: Sensitivity Analysis
STD: Standard deviation
WLC: Weighted Linear Combination
